# Supplementary material for: The interactome of intact mitochondria by cross-linking mass spectrometry provides evidence for coexisting respiratory supercomplexes
Source: Mol Cell Proteomics. 2017 Dec 8;17(2):216–32. doi: 10.1074/mcp.RA117.000470 (PMC5795388; doi:10.1074/mcp.RA117.000470)
Supplement: Supplemental Data [file supp_RA117.000470_133922_0_supp_23978_fzffwf.zip › spectra_annotation/mito_spectra_annotation/257-1-4-1-10-1.pdf]

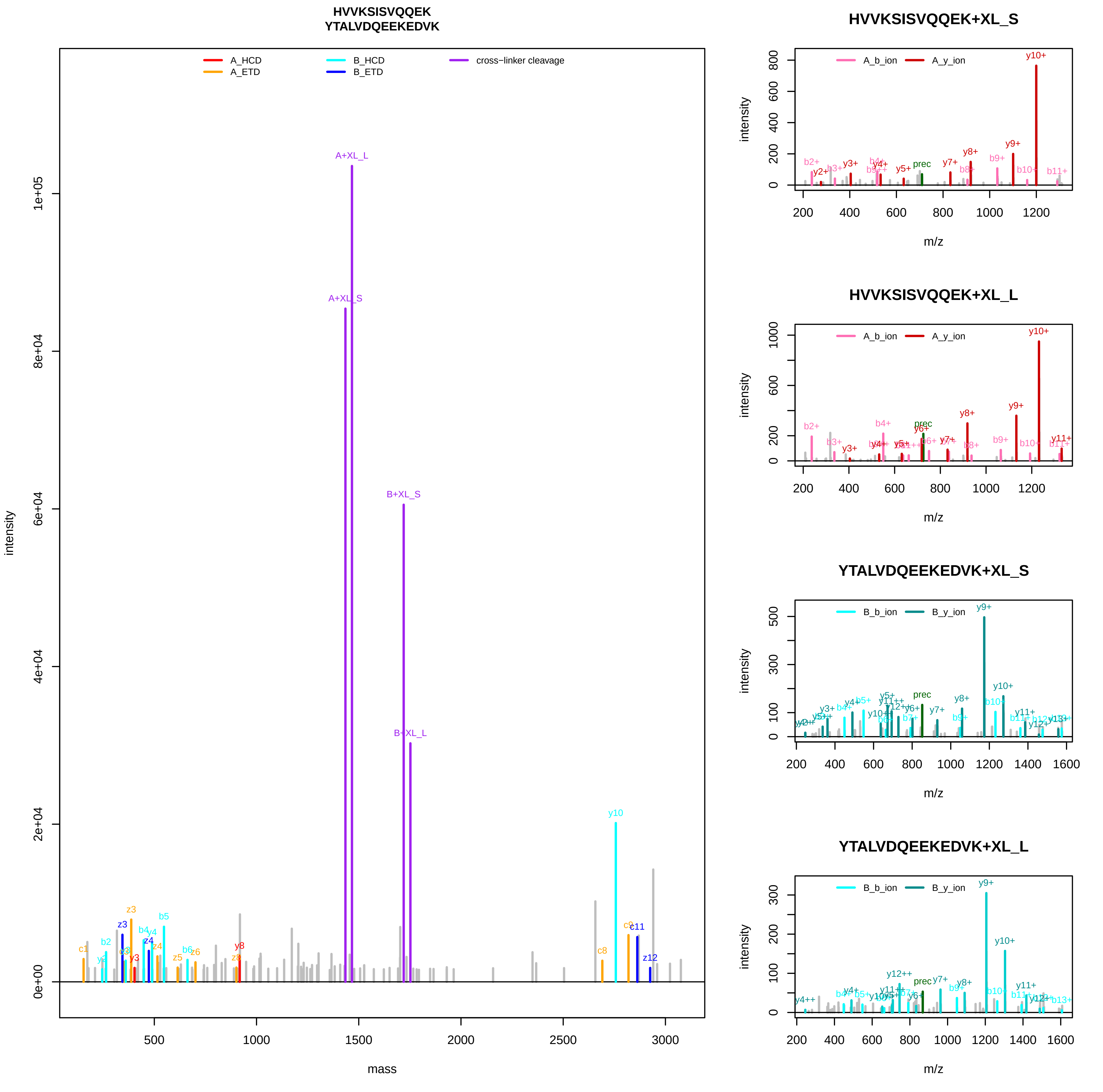

HVVKSISVQQEK
YTALVDQEEKEDVK
A_HCD
A_ETD
B_HCD
B_ETD
cross−linker cleavage
A+XL_L
A+XL_S
B+XL_S
B+XL_L
y10
c9
c11
z12
c8
z3
b5
z4
y8
z8
b2
c1
intensity
mass
0e+00
2e+04
4e+04
6e+04
8e+04
1e+05
500
1000
1500
2000
2500
3000
HVVKSISVQQEK+XL_S
A_b_ion
A_y_ion
y10+
y9+
b9+
y8+
y7+
prec
y5+
y3+
b2+
y2+
b3+
b10+
b11+
HVVKSISVQQEK+XL_L
A_b_ion
A_y_ion
y10+
y9+
y8+
b4+
y6+
prec
y7+
y11+
b11+
YTALVDQEEKEDVK+XL_S
B_b_ion
B_y_ion
y9+
y10+
prec
y8+
y7+
y11+
b5+
y4+
y3+
YTALVDQEEKEDVK+XL_L
B_b_ion
B_y_ion
y9+
y10+
y12++
prec
y7+
y8+
b9+
y11+
intensity
m/z
200
400
600
800
1000
1200
1400
1600
